# Supplementary material for: Examining pathways between structural stigma and tobacco use: a comparison among young adults living in the United States by sexual orientation and gender identity
Source: Int J Equity Health. 2025 May 8;24:128. doi: 10.1186/s12939-025-02487-2 (PMC12060347; doi:10.1186/s12939-025-02487-2)
Supplement: Supplementary file 1 — Supplementary Material 1 [file 12939_2025_2487_MOESM1_ESM.docx]

Supplementary Table 1. Breakdown by state of structural stigma variables

|  | n | Anti-gay prejudice at the state-level in 2021  (0-5 scale) | Absence of state-level LGBTQ protective policies in 2022  (0-10 scale) | Presence of state-level LGBTQ discriminatory policies introduced or enacted in 2022  (0-20 scale) | Total Score  (0-35) |
| --- | --- | --- | --- | --- | --- |
| Alabama | 43 | 4 | 10 | 3 | **17** |
| Alaska | 3 | 2 | 9 | 1 | **12** |
| Arizona | 54 | 2 | 9 | 8 | **19** |
| Arkansas | 17 | 5 | 10 | 0 | **15** |
| California | 296 | 2 | 2 | 0 | **4** |
| Colorado | 37 | 2 | 0 | 0 | **2** |
| Connecticut | 18 | 1 | 0 | 0 | **1** |
| Delaware | 7 | 4 | 2 | 1 | **7** |
| District of Columbia | 9 | 4 | 2 | 0 | **6** |
| Florida | 179 | 4 | 10 | 4 | **18** |
| Georgia | 99 | 4 | 10 | 3 | **17** |
| Hawaii | 9 | 2 | 2 | 1 | **5** |
| Idaho | 5 | 2 | 10 | 1 | **13** |
| Illinois | 126 | 2 | 0 | 1 | **3** |
| Indiana | 49 | 2 | 8 | 6 | **16** |
| Iowa | 14 | 1 | 0 | 8 | **9** |
| Kansas | 12 | 1 | 8 | 2 | **11** |
| Kentucky | 50 | 4 | 8 | 5 | **17** |
| Louisiana | 41 | 5 | 10 | 4 | **19** |
| Maine | 13 | 1 | 0 | 0 | **1** |
| Maryland | 45 | 4 | 2 | 1 | **7** |
| Massachusetts | 47 | 1 | 0 | 1 | **2** |
| Michigan | 80 | 2 | 8 | 3 | **13** |
| Minnesota | 37 | 1 | 0 | 2 | **3** |
| Mississippi | 14 | 4 | 10 | 5 | **19** |
| Missouri | 38 | 1 | 9 | 3 | **13** |
| Montana | 5 | 2 | 8 | 0 | **10** |
| Nebraska | 13 | 1 | 10 | 0 | **11** |
| Nevada | 30 | 2 | 0 | 0 | **2** |
| New Hampshire | 9 | 1 | 2 | 3 | **6** |
| New Jersey | 53 | 1 | 0 | 4 | **5** |
| New Mexico | 8 | 2 | 0 | 0 | **2** |
| New York | 164 | 1 | 0 | 0 | **1** |
| North Carolina | 105 | 4 | 8 | 2 | **14** |
| North Dakota | 11 | 1 | 10 | 0 | **11** |
| Ohio | 143 | 2 | 8 | 2 | **12** |
| Oklahoma | 24 | 5 | 10 | 8 | **23** |
| Oregon | 31 | 2 | 2 | 0 | **4** |
| Pennsylvania | 139 | 1 | 8 | 2 | **11** |
| Rhode Island | 10 | 1 | 0 | 2 | **3** |
| South Carolina | 37 | 4 | 10 | 9 | **23** |
| South Dakota | 2 | 1 | 10 | 3 | **14** |
| Tennessee | 70 | 4 | 10 | 4 | **18** |
| Texas | 239 | 5 | 10 | 0 | **15** |
| Utah | 21 | 2 | 4 | 3 | **9** |
| Vermont | 2 | 1 | 0 | 0 | **1** |
| Virginia | 78 | 4 | 0 | 4 | **8** |
| Washington | 63 | 2 | 0 | 0 | **2** |
| West Virginia | 14 | 4 | 10 | 5 | **19** |
| Wisconsin | 42 | 2 | 5 | 2 | **9** |
| Wyoming | 4 | 2 | 10 | 2 | **14** |
