# Supplementary material for: Examining pathways between structural stigma and tobacco use: a comparison among young adults living in the United States by sexual orientation and gender identity
Source: Int J Equity Health. 2025 May 8;24:128. doi: 10.1186/s12939-025-02487-2 (PMC12060347; doi:10.1186/s12939-025-02487-2)
Supplement: Supplementary file 2 — Supplementary Material 2 [file 12939_2025_2487_MOESM2_ESM.docx]

Supplemental Table 2. Sexual orientation and gender identity breakdown within the SGM YA group (N = 1,281)

| Sexual Orientation | Gender | N | % |
| --- | --- | --- | --- |
| Gay | Cisgender male | 119 | 9.3 |
| Lesbian | Cisgender female | 104 | 8.1 |
| Lesbian/Gay | Transgender/non-binary | 62 | 4.8 |
| Bisexual | Cisgender male | 209 | 16.3 |
| Bisexual | Cisgender female | 522 | 40.7 |
| Bisexual | Transgender/non-binary | 150 | 11.7 |
| Other^1^ | Cisgender male | 27 | 2.1 |
| Other | Cisgender female | 57 | 4.4 |
| Other | Transgender/non-binary | 31 | 2.4 |

1.The other category was followed by a write in option which included orientations such as pansexual, demisexual, asexual, and plurisexual.
